# Overexpression of rice jacalin-related mannose-binding lectin (OsJAC1) enhances resistance to ionizing radiation in *Arabidopsis*

**DOI:** 10.1186/s12870-019-2056-8

**Published:** 2019-12-18

**Authors:** In Jung Jung, Joon-Woo Ahn, Sera Jung, Jung Eun Hwang, Min Jeong Hong, Hong-Il Choi, Jin-Baek Kim

**Affiliations:** 1 Advanced Radiation Technology Institute, Korea Atomic Energy Research Institute, 29 Geumgu-gil, Jeongeup-si, Jeollabuk-do 56212 Republic of Korea; 2 Division of Ecological Conservation, Bureau of Ecological Research, National Institute of Ecology, Seocheon, 33657 Republic of Korea

**Keywords:** Jacalin-related lectin (JRL), Ionizing radiation, Transcriptome analysis, DNA damage response (DDR)

## Abstract

**Background:**

Jacalin-related lectins in plants are important in defense signaling and regulate growth, development, and response to abiotic stress. We characterized the function of a rice mannose-binding jacalin-related lectin (OsJAC1) in the response to DNA damage from gamma radiation.

**Results:**

Time- and dose-dependent changes of *OsJAC1* expression in rice were detected in response to gamma radiation. To identify OsJAC1 function, OsJAC1-overexpressing transgenic *Arabidopsis* plants were generated. Interestingly, OsJAC1 overexpression conferred hyper-resistance to gamma radiation in these plants. Using comparative transcriptome analysis, genes related to pathogen defense were identified among 22 differentially expressed genes in OsJAC1-overexpressing *Arabidopsis* lines following gamma irradiation. Furthermore, expression profiles of genes associated with the plant response to DNA damage were determined in these transgenic lines, revealing expression changes of important DNA damage checkpoint and perception regulatory components, namely *MCMs*, *RPA*, *ATM*, and *MRE11*.

**Conclusions:**

OsJAC1 overexpression may confer hyper-resistance to gamma radiation via activation of DNA damage perception and DNA damage checkpoints in *Arabidopsis,* implicating OsJAC1 as a key player in DNA damage response in plants. This study is the first report of a role for mannose-binding jacalin-related lectin in DNA damage.

## Background

Lectins are carbohydrate-binding proteins that play diverse roles in both plants and animals [1]. In plants, lectins interact with endogenous carbohydrates and reportedly are involved in signaling pathways [2]. Twelve subfamilies of plant lectins have been identified [3]. One subfamily, the jacalin-related lectins (JRLs), is named for the presence of a jacalin-like domain and comprises 25 identified members [4]. This large subfamily has been further divided into two subgroups, based on the members’ carbohydrate-binding properties, subcellular localization, and molecular structures [5]. For example, mannose-binding JRLs are located in both the nucleus and the cytosol, whereas galactose-binding JRLs are located in vascular compartments [5]. Plant JRLs are important in the response to biotic stresses, such as pathogen and insect attack [6], as well as abiotic stresses, such as salinity stress [7]. Functionally, most JRLs are related to disease resistance and signaling in response to multiples stresses [8]. Particularly, JRLs with dirigent domains have been associated with plant defenses to pathogens. OsJAC1 is a mannose-binding JRL from rice (*Oryza sativa*). This factor contains a dirigent domain in its N-terminal region as described by Jiang et al. [9]. Overexpression of OsJAC1 suppressed elongation of coleoptiles and internodes, consistent with a regulatory function for OsJAC1 in growth and development [10]. Furthermore, Weidenbach et al. [11] concluded that this protein is also involved in plant defense to pathogen attack.

The genomes of all organisms are vulnerable to a variety of detrimental endogenous and exogenous factors, including replication errors, reactive oxygen species (ROS), ionizing radiation, and genotoxic chemicals. Ionizing radiation, which includes gamma radiation, is a carcinogen. Gamma irradiation directly damages a genome by introducing double-strand breaks (DSBs) in the DNA [12]. Repair of DSBs occurs via two important pathways: non-homologous end joining and homologous recombination [13]. In addition, gamma radiation also indirectly induces DNA damage via the generation of ROS, which introduces different types of DNA lesions [14]. Cellular DNA damage response (DDR) mechanisms, including repair mechanisms, to maintain genomic integrity, are fundamentally conserved across all organisms [15, 16]. One important regulator of DDR is ataxia telangiectasia mutated (ATM) protein [17], which is a signal transducer that acts in response to DSBs. Ataxia telangiectasia and RAD3-related (ATR) protein is also involved in signaling in response to single-strand breaks and stalled replication forks [18].

DNA replication is important for transmission of genetic information to daughter cells and progeny; therefore, all organisms have mechanisms to protect the fidelity of DNA replication. For example, DNA damage can adversely affect the replication machinery and result in a stalled replication fork. DNA replication is initiated at numerous origins of replication in eukaryotes [19] via a two-step process. The first step is origin licensing, which starts with a pre-replicative complex in late mitosis or the G1 phase of the cell cycle [20]. The pre-replicative complex is composed of cell division 6 (CDC6), the origin-recognition complex, the cell division cycle 10-dependent transcript 1 (Cdt1), and mini-chromosome maintenance proteins 2–7 (MCM2-MCM7). The second step, origin firing, begins with activation of the MCM2–7 complex. Component kinases, such as cycle dependent kinase (CDK) and Dbf-dependent kinase (DDK), that are specific to the S phase of the cell cycle are required for this origin firing step [20, 21].

In our preliminary microarray studies, differential expression of *OsJAC1* was found in response to ionizing radiation (unpublished data). Several studies reported that plant JRLs are involved in responses to abiotic and biotic stress [6–8]; however, no evidence for a role of JRLs in DDR has been published. Therefore, we examined the molecular function of OsJAC1 in DDR. We sought to establish the effect of ionizing radiation and abiotic stresses on the expression of *OsJAC1.* We also generated transgenic OsJAC1-overexpressing *Arabidopsis* lines that were resistant to gamma irradiation. We probed the molecular mechanism underlying OsJAC1 function on DDR using comparative transcriptome analysis of the OsJAC1-overexpressing lines.

## Results

### Expression analysis of *OsJAC1* in rice plants in response to ionizing radiation, abiotic stresses, and plant hormones

**Fig. 1:**
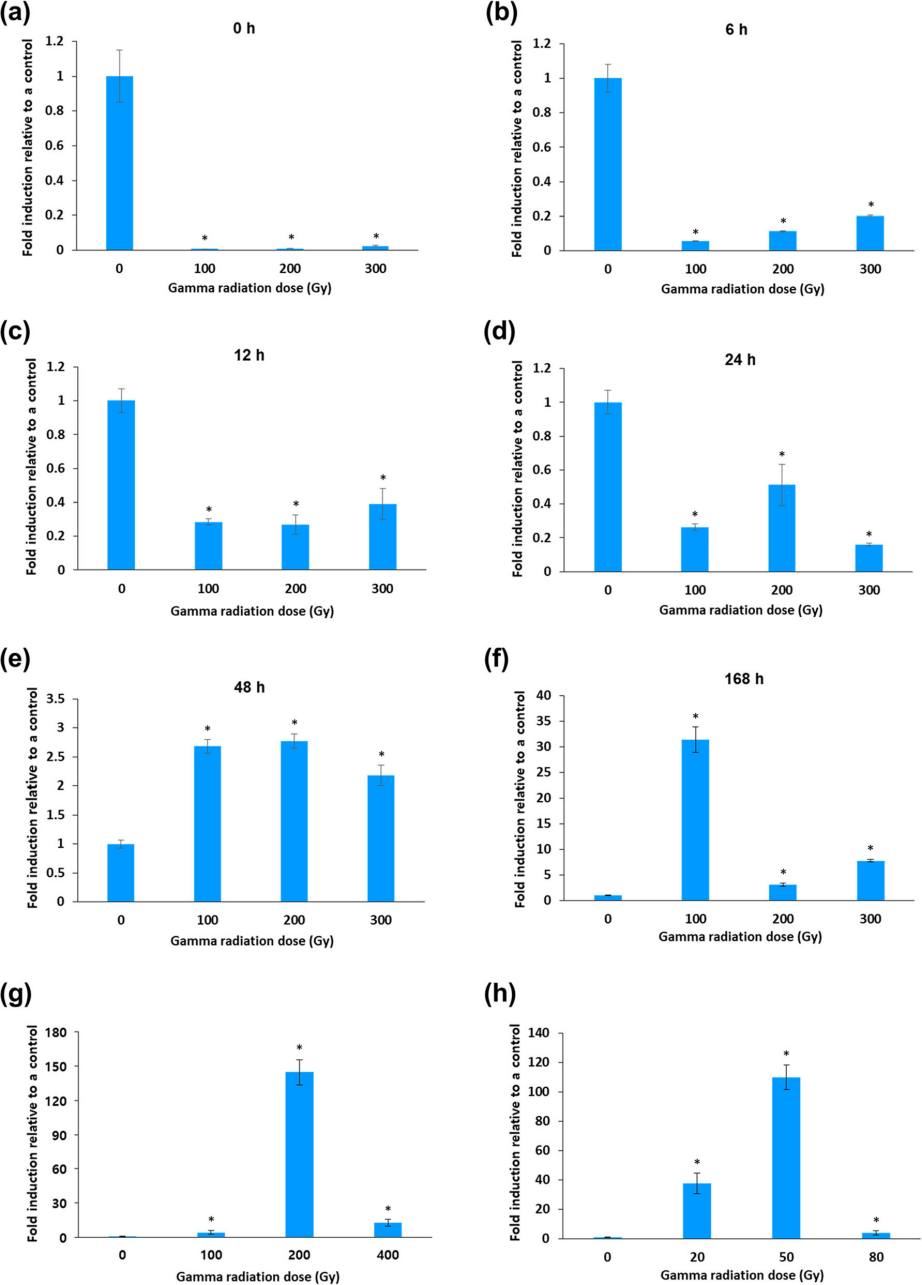
Expression of *OsJAC1* in rice seedlings irradiated with ionizing radiation as determined with quantitative RT-PCR. **a**-**f**: Time courses of expression of *OsJAC1* in 2-week-old rice seedlings after exposure to the indicated levels of gamma radiation. **g**, **h**: Expression of *OsJAC1* in 2-week-old seedlings from rice seeds that had been irradiated with gamma radiation (**g**) or with an ion beam (**h**) and then germinated on MS media. Values represent means ± SD (*n* = 3). Statistical analysis was carried out by one-way ANOVA (*p < 0.01)

We measured *OsJAC1* expression over time in 2-week-old seedlings after exposure to different dosages of gamma radiation. *OsJAC1* expression was greatly reduced in rice seedlings immediately after exposure at all levels of irradiation tested (Fig. 1a). Compared to untreated controls, the numbers of *OsJAC1* transcripts were reduced approximately 150- and 50-fold in plants exposed to 100 and 300 Gy gamma irradiation, respectively. The transcript levels were slightly increased 6, 12, and 24 h after irradiation compared to the 0-h time point (Fig. 1b-d); however, by 48 h after irradiation, we observed a greater than 2-fold induction of *OsJAC1* expression in seedlings compared to levels in a non-irradiated control (Fig. 1e). Furthermore, the numbers of transcripts were increased at all doses of irradiation at 168 h (corresponding to 7 d) compared to the unirradiated control. These increases were approximately 30-, 4-, and 8-fold at 100, 200, and 300 Gy of gamma irradiation, respectively (Fig. 1f). To confirm this late induction of *OsJAC1* transcript expression in response to ionizing radiation, dry rice seeds were irradiated with gamma radiation or an ion beam, subsequently germinated on MS media, and irradiated after 2 weeks. These seedlings exhibited increased *OsJAC1* transcripts in response to both types of radiation (Fig. 1g, h).

**Fig. 2:**
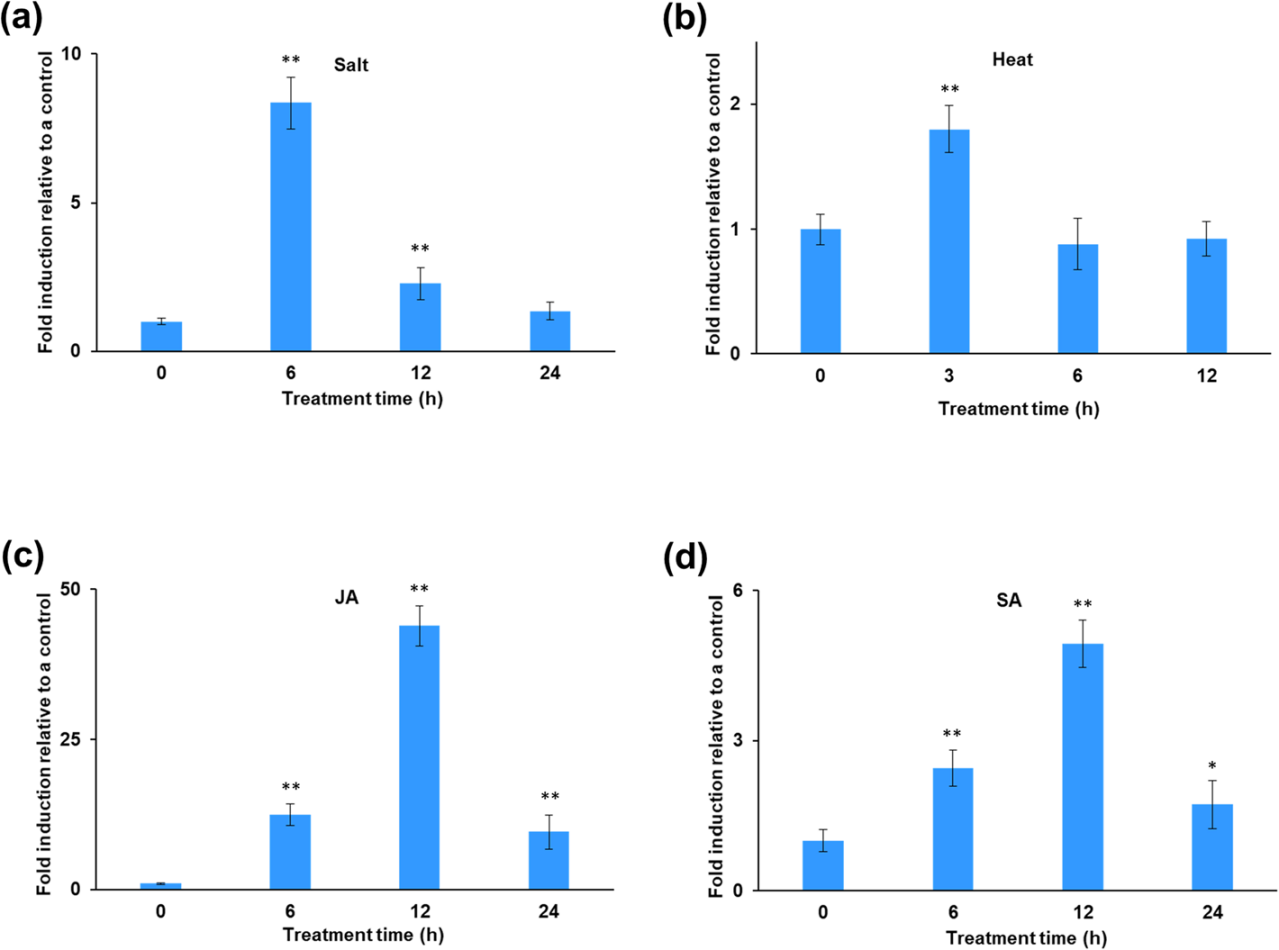
Time course of expression of *OsJAC1* in 2-week-old rice seedlings exposed to abiotic stresses (**a**) salinity stress or (**b**) heat stress or to plant hormones (**c**) SA or (**d**) JA as determined by quantitative RT-PCR. Data represent means ± SD (n = 3). One-way ANOVA was used for statistical analysis (**p < 0.01, 0.01 < *p < 0.05)

Additionally, *OsJAC1* expression was altered by exposure to other stressors. *OsJAC1* expression was also upregulated in response to salinity stress (Fig. 2a). In seedlings treated with NaCl for 6 h, we observed an approximately 8-fold increase in the number of *OsJAC1* transcripts compared to untreated seedlings. The *OsJAC1* transcript expression was also slightly increased after 3 h of exposure to heat stress, although no significant difference was observed after 6 or 12 h of exposure (Fig. 2b). Expression levels of *OsJAC1* were also upregulated by jasmonic acid (JA) and salicylic acid (SA) treatment (Fig. 2c, d). *OsJAC1* expression was approximately 40-fold higher 12 h after JA treatment, while SA treatment resulted in a 5-fold induction of *OsJAC1* expression at this time point compared with levels in the untreated control.

### Generation of *Arabidopsis* OsJAC1-overexpressing lines

**Fig. 3:**
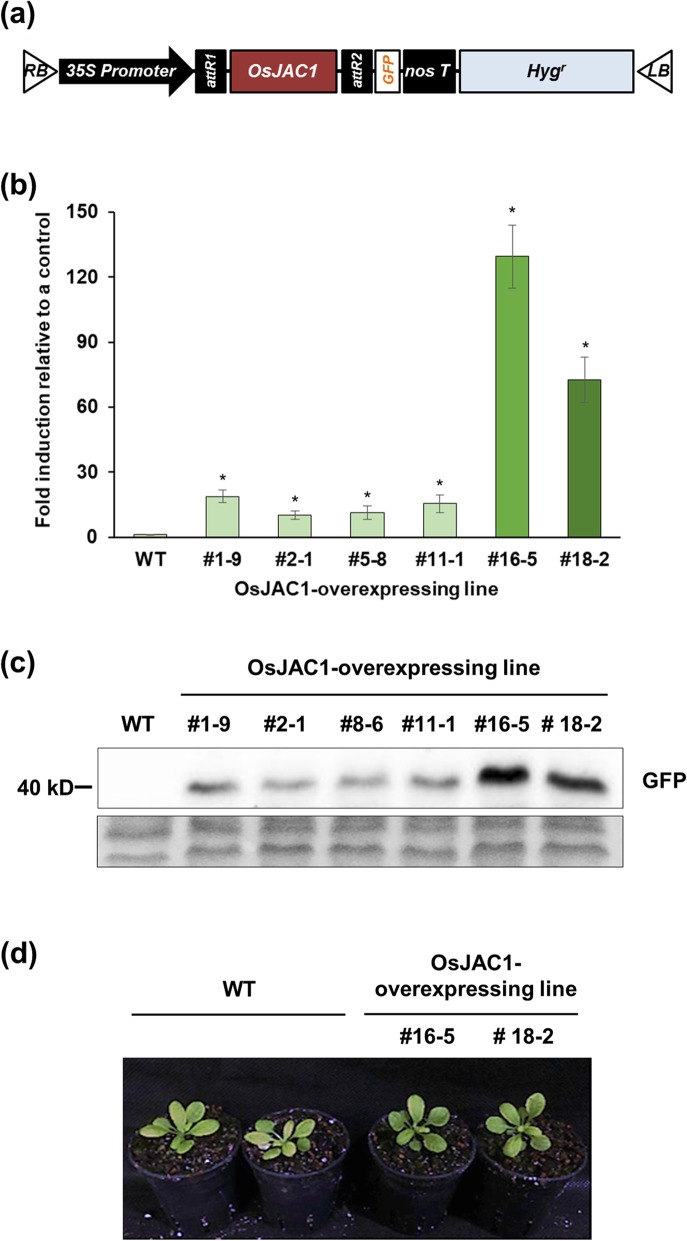
Generation of OsJAC1-overexpressing *Arabidopsis* lines and confirmation of enhanced expression. **a** Schematic diagram of vector construct for *OsJAC1* overexpression. **b** *OsJAC1* transcripts in *OsJAC1*-overexpressing lines were detected using quantitative RT-PCR. Data represent means ± SD (n = 3). Statistical analysis was carried out by one-way ANOVA (*p < 0.01). **c** Expression levels of *OsJAC1* in *OsJAC1*-overexpressing lines as determined using western blot. **d** Photographs of *OsJAC1*-overexpressing lines and wild-type plants 30 d after sowing. Note that morphologies are similar

We next sought to probe the molecular function of OsJAC1 by generating OsJAC1-overexpressing *Arabidopsis* lines. A schematic diagram (Fig. 3a) shows the structure of the OsJAC1-overexpressing construct in which *OsJAC1* is regulated by the *35S* promoter and terminator. Two transgenic lines, #16–6 and #18–2, displayed significant overexpression, approximately 70- and 130-fold, respectively (Fig. 3b). *OsJAC1* overexpression was accompanied by higher levels of OsJAC1 protein in both transgenic lines than in a wild-type control (Fig. 3c). Figure 3d displays the morphology of the transgenic lines in the early vegetative growth stage, revealing no obvious morphological differences in the transgenic lines in comparison to a wild-type control in the absence of exposure to radiation.

### OsJAC1 overexpression leads to hyper-resistance to gamma radiation

**Fig. 4:**
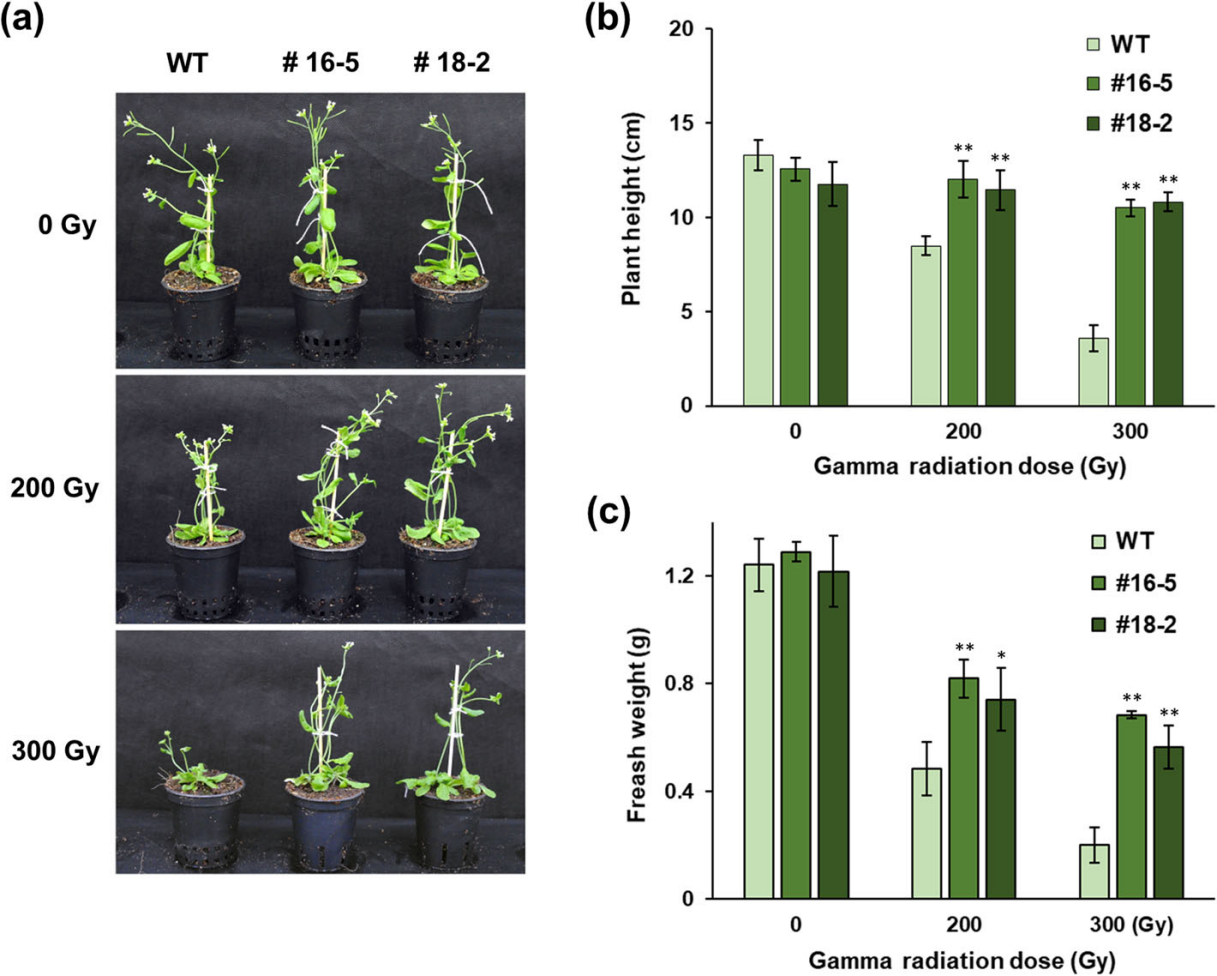
Morphological features and growth responses of OsJAC1-overexpressing *Arabidopsis* lines in response to gamma radiation. **a** Two-week-old seedlings were irradiated using gamma radiation. Photographs of *OsJAC1*-overexpressing lines and wild-type plants 30 d after irradiation. **b**, **c** Heights and fresh weights of *OsJAC1*-overexpressing lines and wild-type plants after gamma irradiation. Data represent means ± SD (n = 3). Statistical analysis was carried out by one-way ANOVA (**p < 0.01, 0.01 < *p < 0.05)

We then assessed the effect of OsJAC1 overexpression on growth and development in response to gamma radiation. Transgenic lines and wild-type control plants were irradiated with 200 or 300 Gy gamma radiation, and growth rates were compared 2 weeks later. There were no morphological differences between the transgenic and control plants in the reproductive stage in the absence of irradiation (Fig. 4a). Following irradiation, the OsJAC1-overexpressing lines grew faster than wild-type plants at both doses of irradiation (Fig. 4a). Consequently, the overexpressing lines were taller and accumulated more mass than the irradiated control plants (Fig. 4b, c). Specifically, both OsJAC1-overexpressing lines displayed plant heights and fresh weights that were more than 3-fold higher than those in controls after treatment with 300 Gy gamma radiation. We also measured the growth rates of OsJAC1-overexpressing lines treated with NaCl as a means to impose salinity stress. OsJAC1 overexpression enhanced root growth in the stressed plants compared to unstressed plants (Additional file 1: Figure S1). Therefore, we conclude that plants with OsJAC1 overexpression possess resistances to both gamma radiation and salinity stress.

### Transcriptomic analysis of the DNA damage response in OsJAC1-overexpressing lines

**Fig. 5:**
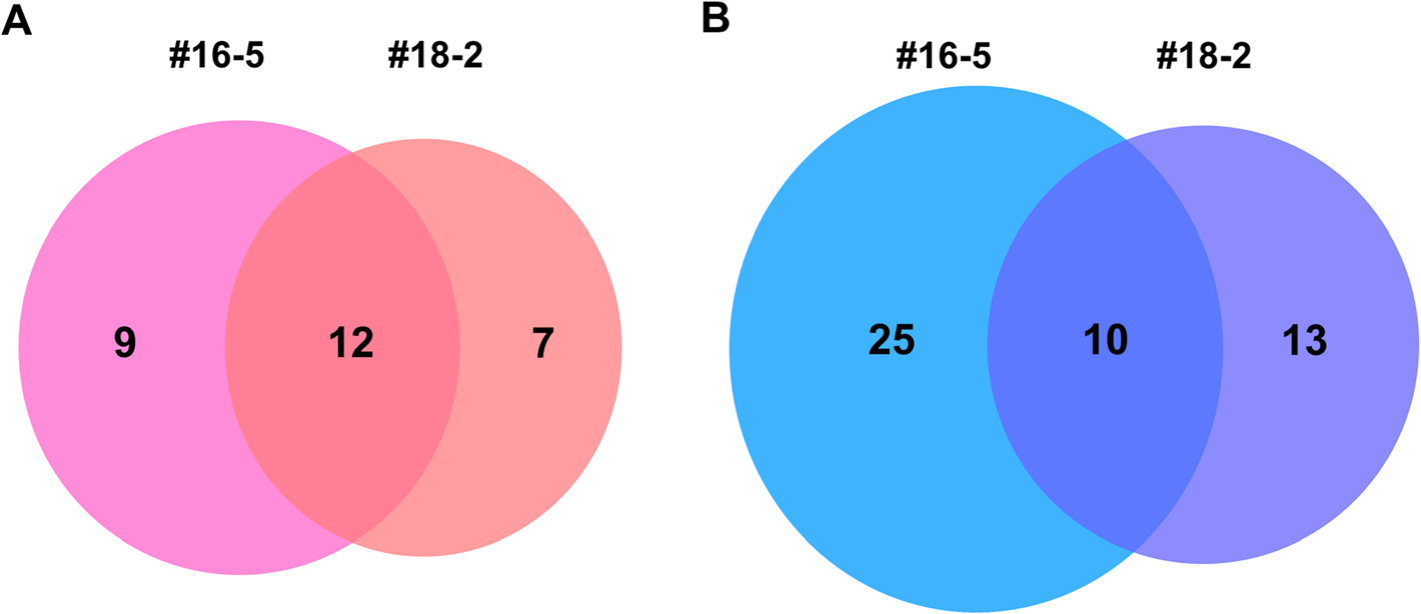
DEG analysis of OsJAC1-overexpressing *Arabidopsis* lines compared to a wild-type control after 100 Gy gamma irradiation. Venn diagrams show number of upregulated (**a**) and downregulated (**b**) DEGs

Our next step was to probe the molecular function of OsJAC1 in DDR. We performed transcriptome analysis of OsJAC1-overexpressing lines. A total of more than 129 million trimmed reads were generated from a wild-type control and two OsJAC1-overexpressing transgenic lines treated with or without gamma irradiation (Table 1). Trimmed reads were mapped to the reference gene set from the ARAPORT database (https://www.araport.org/). The average mapped rate of six samples was 84% (Table 1). Figure 5 shows the number of upregulated and downregulated DEGs in both OsJAC1-overexpressing lines compared to the wild-type control after 100 Gy gamma irradiation. The two transgenic lines shared 12 upregulated and 10 downregulated DEGs. In upregulated DEGs, three xyloglucan endotransglucosylase/hydrolase genes (AT4G14130, AT3G23730, and AT5G65730) were detected (Table 2). Interestingly, pathogen defense-related genes, such as disease resistance proteins (AT5G41740 and AT5G41750) and NPR1-like protein (AT5G45110), were among the downregulated DEGs of both OsJAC1-overexpressing lines. Additional file 2: Table S1 shows expression data for all annotated transcripts in OsJAC1-overexpressing lines..

**Table 1:** Number of trimmed and mapped reads of wild-type and OsJAC1-overexpressing transgenic lines with/without gamma irradiation

Sample	Total trimmed reads^a^	Mapped read	Mapped rate (%)
WT	23,191,133	19,396,927	83.6
16–5	25,199,270	21,188,297	84.0
18–2	20,500,887	18,441,540	89.9
WT (100 Gy)	19,967,350	16,002,371	80.1
16–5 (100 Gy)	19,971,641	17,370,030	86.9
18–2 (100 Gy)	21,120,649	16,840,320	79.7
Total	129,950,930	109,239,485	84.0

^a^ All trimmed reads were summed from the two biological replicates of each sample

**Table 2:** Up- and down-regulated DEGs were commonly detected in both OsJAC1-overexpressing lines

	Locus	Fold induction		Definition
		#16–5	#18–2	
Up	AT4G14120	2.56	2.08	Unknown
	AT4G14130	1.75	1.89	Xyloglucan endotransglucosylase/hydrolase 15
	AT3G23730	1.57	1.54	Xyloglucan endotransglucosylase/hydrolase 16
	AT2G30600	1.28	1.51	BTB/POZ domain-containing protein
	AT5G44130	1.22	1.19	FASCICLIN-like arabinogalactan protein 13 precursor
	AT2G17230	1.18	1.11	EXORDIUM like 5
	AT4G25580	1.12	1.11	CAP160 protein
	AT3G19680	1.11	1.04	Protein of unknown function (DUF1005)
	AT4G16563	1.11	1.35	Eukaryotic aspartyl protease family protein
	AT5G46760	1.08	1.18	Basic helix-loop-helix (bHLH) DNA-binding family protein
	AT5G46750	1.05	1.01	ARF-GAP domain 9
	AT5G65730	1.01	1.01	Xyloglucan endotransglucosylase/hydrolase 6
Down	AT5G47910	−1.83	−1.47	Respiratory burst oxidase homologue D
	AT5G41750	−1.76	−1.70	Disease resistance protein (TIR-NBS-LRR class) family
	AT5G41740	−1.67	−1.69	Disease resistance protein (TIR-NBS-LRR class) family
	AT4G34150	−1.23	−1.20	Calcium-dependent lipid-binding (CaLB domain) family protein
	AT5G35735	−1.22	−1.46	Auxin-responsive family protein
	AT1G61890	−1.19	−1.50	MATE efflux family protein
	AT2G38470	−1.06	−1.10	WRKY DNA-binding protein 33
	AT5G45110	−1.06	−1.22	NPR1-like protein 3
	AT4G29780	−1.05	−1.93	Unknown
	AT4G33920	−0.62	−1.00	Protein phosphatase 2C family protein

**Fig. 6:**
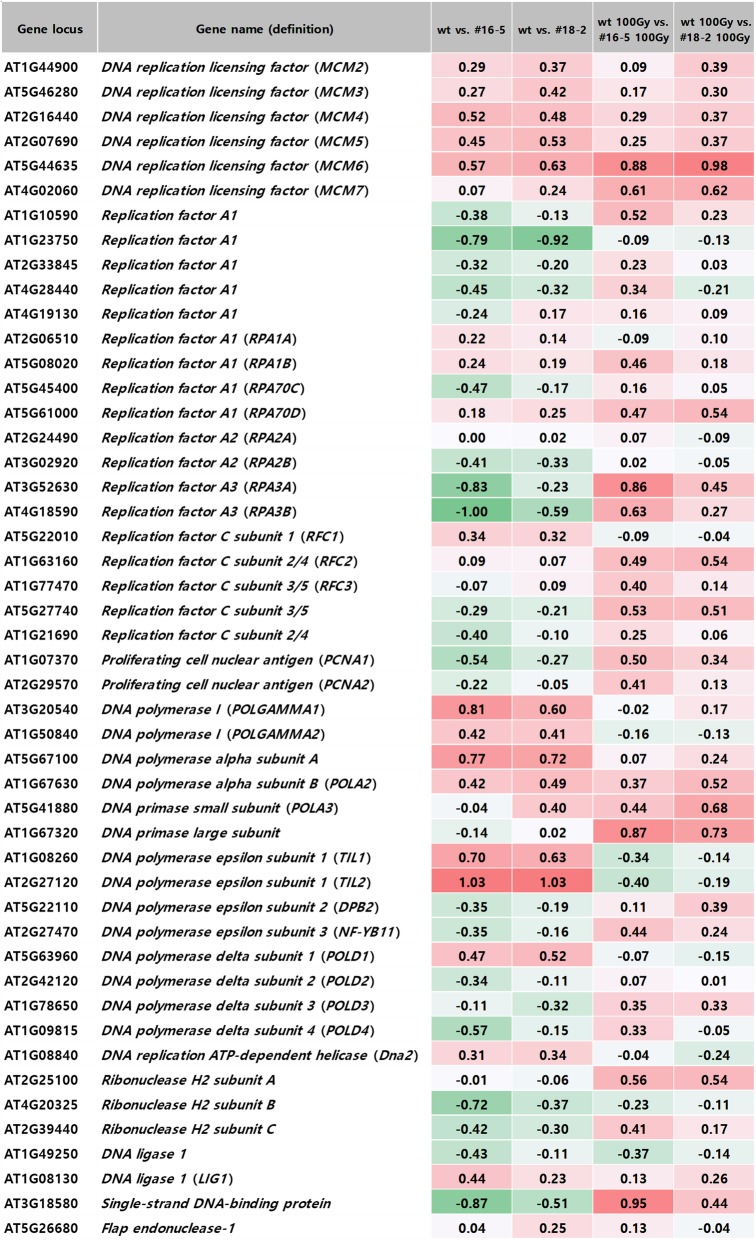
Comparative transcriptome expression profiles of genes involved in DNA replication from *OsJAC1*-overexpressing lines and a wild-type control before and after gamma irradiation

We next assessed the expression profile of genes involved in DNA replication in OsJAC1-overexpressing lines with and without gamma irradiation (Fig. 6). In the absence of irradiation, expression of *MCM5*, *6*, and *7* was greater in OsJAC1-overexpressing lines than in the wild-type control. Following irradiation, the expression of *MCM6* and *MCM7* was significantly upregulated in OsJAC1-overexpressing lines compared to the irradiated control plant.

Additionally, the transcript level of At1g23750 (replication protein A1) was significantly reduced by OsJAC1 overexpression in the absence of irradiation compared to the wild-type control*.* There were fewer *RPA3A* and *RPA3B* transcripts in *OsJAC1*-overexpressing lines without gamma irradiation compared to the wild-type control, whereas gamma irradiation resulted in transcriptional induction of these two genes (Fig. 6). Both *POLGAMMA1* and the At5g67100 (DNA polymerase alpha subunit A) gene were upregulated in the transgenic lines in the absence of irradiation compared to the wild-type plants. Similarly, the expression levels of polymerase epsilon subunits *TIL1* and *TIL2* were increased by OsJAC1 overexpression under non-irradiated conditions, whereas slight reductions of these transcripts were observed after gamma irradiation. In addition, gamma irradiation resulted in transcriptional induction of the At1g67320 (DNA primase large subunit) gene in the transgenic lines (Fig. 6).

**Fig. 7:**
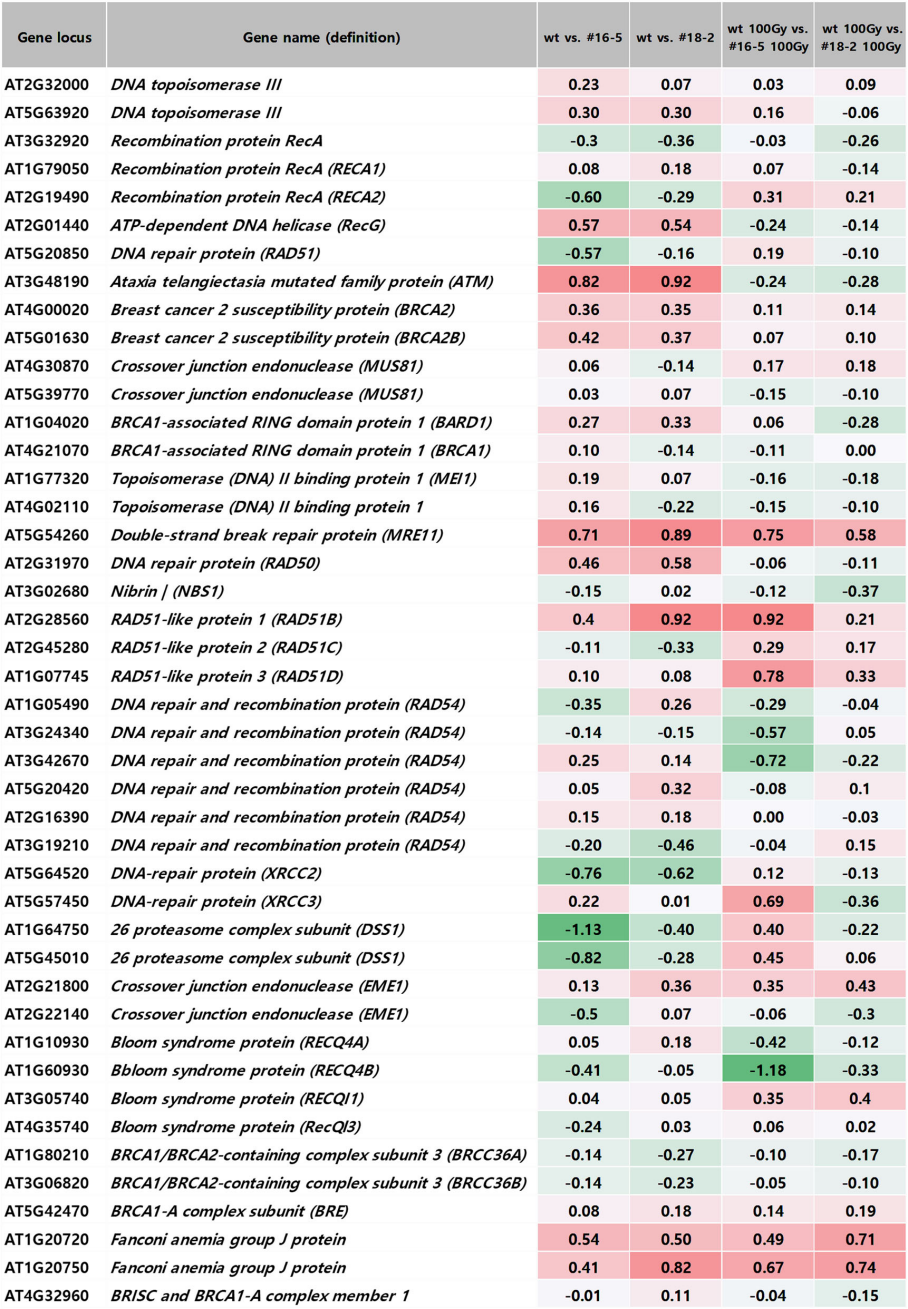
Comparative transcriptome expression profiles of genes associated with homologous recombination from *OsJAC1*-overexpressing lines and a wild-type control with and without gamma irradiation

Figure 7 displays the expression levels of genes involved in homologous recombination repair. OsJAC1 overexpression affected the accumulation of *ATM*. Expression of this gene was significantly upregulated in non-irradiated OsJAC1-overexpressing lines compared to the wild-type control. Interestingly, we did not detect significant differences in *ATR* expression between the overexpressing lines and the wild-type control (data not shown). *Meiotic recombination 11* (*MRE11*) and *Fanconi anemia group J protein* were upregulated by OsJAC1 overexpression in both irradiated and non-irradiated plants (Fig. 7).

**Fig. 8:**
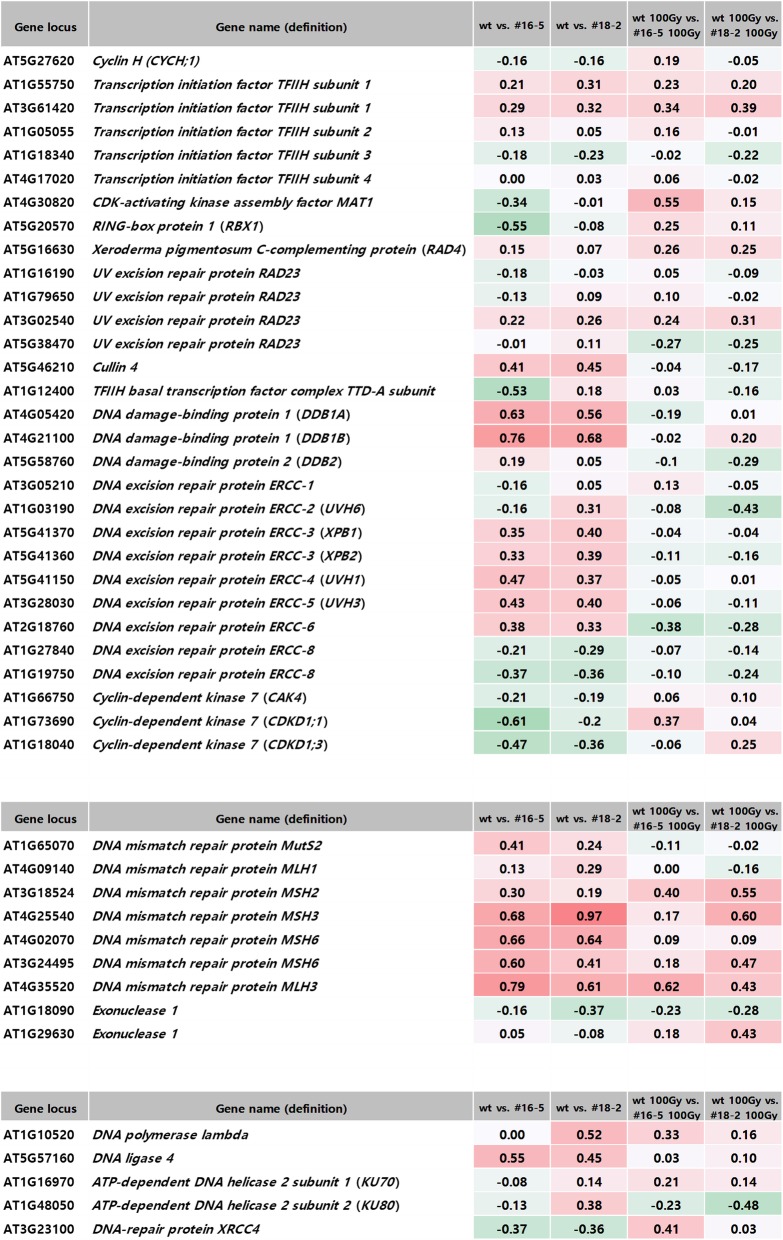
Comparative transcriptome expression profiles for genes related to (a) nucleotide excision repair, (b) mismatch repair, and (c) non-homologous recombination repair from *OsJAC1*-overexpressing lines and a wild-type control before and after gamma irradiation

Figure 8 shows the expression patterns of genes related to nucleotide excision repair, mismatch repair, and non-homologous recombination. In nucleotide excision repair, OsJAC1 overexpression enhanced the transcriptional accumulation of *DDB1A* and *DDB1B* (UV-damaged DNA damage-binding proteins) under non-irradiated conditions (Fig. 8a)*.* DNA mismatch repair genes *MSH3*, *MSH6*, and *MLH3* were increased in both transgenic lines (Fig. 8b), and gene expression of the non-homologous recombination repair factor At4G57160 (DNA ligase 4) was increased by OsJAC1 overexpression without gamma irradiation (Fig. 8c).

## Discussion

### OsJAC1 is involved in the response to abiotic stress, including gamma irradiation and salinity stress

JRLs are associated with plant responses to stress, including abiotic stresses and attack by pathogens [8]. The expression of *OsJAC1*, which encodes a JRL, was upregulated in a time- and dose-dependent manner following exposure to both gamma radiation and an ion beam (Fig. 1). We noted some similarities between these responses and two relevant previous studies. Jin et al. [22], using microarray analysis, observed time- and dose-dependent expression of genes associated with signal transduction, transcription, and metabolism in human mesenchymal stem cells exposed to gamma radiation. These genes were either involved in cellular defense, such as apoptosis and responses to stress, or in fundamental cellular processes, such as DNA replication and repair. It has been also been noted that in *Chlamydomonas reinhardtii* [23], the expression of many DDR genes was altered by gamma irradiation. From the similarities between the response of *OsJAC1* and these other genes to radiation, we hypothesized that OsJAC1 may participate in DDR, perhaps in signal transduction involved in these processes.

Given the central role of JRLs in the response of plants to stress, we also examined the response of *OsJAC1* expression to salinity stress. Salinity stress, like irradiation, increased *OsJAC1* expression in rice (Fig. 2a), and OsJAC1-overexpressing lines displayed resistance to salinity stress compared to a wild-type control (Additional file 1: Figure S1). Similar observations were made by Zhang et al. [7], who also identified a relationship between lectins and abiotic stresses, including salinity stress, in rice. One effect of salinity stress in plants is the generation of ROS [24], which are also generated by ionizing radiation. ROS damages cellular components, including DNA, in numerous ways [25, 26], and these similar responses further strengthen the relationship between OsJAC1 and DDR.

JRLs are regulated by the plant hormones JA and SA, which are related to stress responses and pathogen defense in plants [11, 27, 28]. Thus, we examined the effect of these hormones on expression of *OsJAC1.* The hormones enhanced transcription of *OsJAC1* (Fig. 2c, d). SA is associated with genotoxic stress that results from exposure to ethyl methanesulphonate and methyl mercuric chloride [29] and may enhance the genotoxic stress-related signaling pathway [30]; however, the role of SA in this signaling remains unclear [31]. These hormones play central roles in the plant defense response to ROS [32, 33], and their signaling pathways were affected in a dose-dependent manner by H_2_O_2_ accumulation in the *cat2 Arabidopsis* mutant [34, 35]. Similarly, silencing of *mannose-binding lectin* (*CaMLB1*) transcript led to a reduction in both disease resistance and ROS accumulation in pepper plants [36]. Furthermore, Weidenbach et al. [11] reported that OsJAC1 mediated the pathogen defense response in rice. Interestingly, however, DEG analysis displayed downregulation of pathogen defense-related genes in OsJAC1-overexpressing lines (Table 2). These results suggest that OsJAC1 regulates different stresses, such as DNA damage and pathogen attack, via coordination with levels of ROS in plants.

### OsJAC1 overaccumulation leads to modulation of DNA replication components

The relationship between OsJAC1 and abiotic stresses is well documented [7], but the molecular function of this protein has not been established. We first probed the molecular function of OsJAC1 in DDR following exposure of plants to gamma radiation. *Arabidopsis* lines overexpressing OsJAC1 showed tolerance to gamma radiation (Fig. 4). In addition, DEG analysis revealed that these transgenic lines highlighted differential expression of genes involved in pathogen defense after gamma irradiation (Fig. 5 and Table 2). OsJAC1 functions in pathogen defense have been well characterized previously [11]. Hadwiger et al. [37] also reported that DDR is closely associated with pathogen defense via SA signaling. Thus, differential expression of pathogen-related genes in response to gamma radiation in OsJAC1-overexpressing lines indicates that OsJAC1 may function in the overlapping pathways between DDR and pathogen defense.

DDR serves as a regulation signal for many DNA repair pathways, which have presumably evolved to maintain genome integrity. DDR also regulates apoptosis, senescence, and the DNA replication process [38]. DNA replication is a key step for cell proliferation, because genome duplication for transmission is essential in all organisms. Figure 6 shows the expression levels of genes associated with DNA replication in OsJAC1-overexpressing lines. It is of particular interest that the transcript numbers of *MCM4*-*MCM7* were increased in OsJAC1-overexpressing lines. MCM proteins are licensing factors for DNA replication [39]. For formation of the pre-replicative complex, MCMs form a complex with OCR, CDT1, and CDC6/CDC18 [40–42]. *MCM* genes have been identified in *A. thaliana*, *Zea mays*, and *O. sativa* and are expressed in young tissues with replicating cells [39, 43]. We observed significant accumulation of *MCM6* transcripts after exposure to gamma radiation in OsJAC1-overexpressing lines (Fig. 6). Dang et al. [44] noted that the MCM6 single subunit was essential in abiotic stress tolerance in plants. Upregulation of *MCM6* was detected in pea plants exposed to salinity and cold stresses, and overexpression of pea *MCM6* in tobacco conferred resistance to salinity stress. Therefore, upregulation of *MCM* transcripts by OsJAC1 overexpression indicates that OsJAC1 may participate in the regulation of DNA replication stresses induced by salt and gamma radiation.

RPA, which is a single-strand DNA-binding protein that is composed of three subunits (RPA1, 2, 3) is associated with DNA repair, meiosis, and DNA replication and activates cellular responses to DNA damage [45]. Low levels of *RPA3A* and *RPA3B* transcripts were detected in OsJAC1-overexpressing lines before irradiation compared to a wild-type control, but gamma irradiation increased the numbers of these *RPA* transcripts (Fig. 6). DNA polymerase epsilon is composed of four subunits: one large subunit TILl (Pol2) and three small subunits, DNA-binding protein (DPB) 2, 3, and 4 [46]. The exact functions of polymerase delta and epsilon remain controversial, but polymerase epsilon is associated with replicative error repair and replicative stress sensing [47, 48]. In OsJAC1-overexpressing lines, *TIL1* and *TIL2* were upregulated compared to levels in wild-type plants, but genes for both subunits were slightly downregulated following gamma irradiation compared to the levels before irradiation (Fig. 6). *Arabidopsis* mutant *abo4–1*, which has a partially defective polymerase epsilon subunit, was resistant to replicative stress but hypersensitive to DNA damaging agents, including zeiocin [48, 49]. Furthermore, overexpression of polymerase epsilon small subunit DPB2 impaired DNA replication in *Arabidopsis*. Thus, we conclude that OsJAC1 overexpression altered expression of genes involved in DNA replication, implicating OsJAC1 function in DNA replication.

### OsJAC1 may coordinate with MRE11 and ATM to enhance DNA repair

Cellular response to DNA damage is regulated the protein kinases ATM and ATR, which are activated by different types of DNA damage [50–52]. ATM is mainly activated in response to DSBs, while ATR is activated in response to stalled replication forks. Canman et al. [53] observed ATM activation in response to DSB-inducing ionizing radiation in mammalian cells. In the present study, OsJAC1-overexpressing lines exhibited greater *ATM* transcript expression than the wild-type control in the absence of irradiation (Fig. 7), while no difference in the numbers of *ATR* transcripts were observed between the transgenic lines and a control (data not shown). We also observed increased *MRE11* expression in OsJAC1-overexpressing lines compared to the wild-type control (Fig. 7). MRE11 is a component of the MRN complex, which includes radiation sensitive 50 (RAD50) and Nijmegen breakage syndrome 1 (NBS1) and serves as the sensor of DSBs. This complex is also important in DNA damage repair, DNA replication, meiosis, and genome stability [54]. Following binding to DSBs, the MRN complex activates ATM [55, 56], but this complex is not required for ATR activation [57]. Interactions between MRE11 and DNA replication have been noted. Specifically, MRE11 is necessary for the recovery of hydroxyurea-induced replication stress in HeLa cells, and the MRN complex and RPA co-localized and interacted following treatment with either hydroxyurea or UV light [58]. Taken together, these results suggest that OsJAC1 regulates DNA damage perception and DNA repair as well as in DNA replication via coordination with ATM and MRE11.

Furthermore, we examined the role of OsJAC1 in nucleotide excision repair. The UV-damage DNA-binding protein complex was first reported in human cells. Overexpression of *DDB1A* and *DDB1B* enhanced resistance to UV radiation in *Arabidopsis*, whereas two knock-out mutants, *ddb1a* and *ddb1b,* were susceptible [59, 60]. Our results are consistent with this previous report, as *DDB1A* and *DDB1B* transcripts in *OsJAC1*-overexpressing transgenic lines were increased (Fig. 8a). Mismatched nucleotide bases that result from insertion, deletion and mis-incorporation lead to polymerase mis-incorporation and incorrect recombination of DNA. DNA mismatch repair (MMR) systems detect and repair these mismatched nucleotides, and *Mut* genes play important roles in genome maintenance [61]. MSH (MutS homologs) and MHL (MutL homologs) are highly conserved proteins; although, these factors have diverse cellular functions [62]. In the present study, *Arabidopsis* lines overexpressing OsJAC1 had greater expression of *MSH3*, *MSH6*, and *MHL3* transcripts than the wild-type control (Fig. 8b). Previously, MSH2-deficient mouse cells were found to have low survival rates after X-ray irradiation, and MSH2 required re-localization of RAD51 and MRE11 in the G2 phase of the cell cycle [63]. Together, these results may indicate that OsJAC1 is linked with both MMR and NER in the DDR pathway.

## Conclusions

**Fig. 9:**
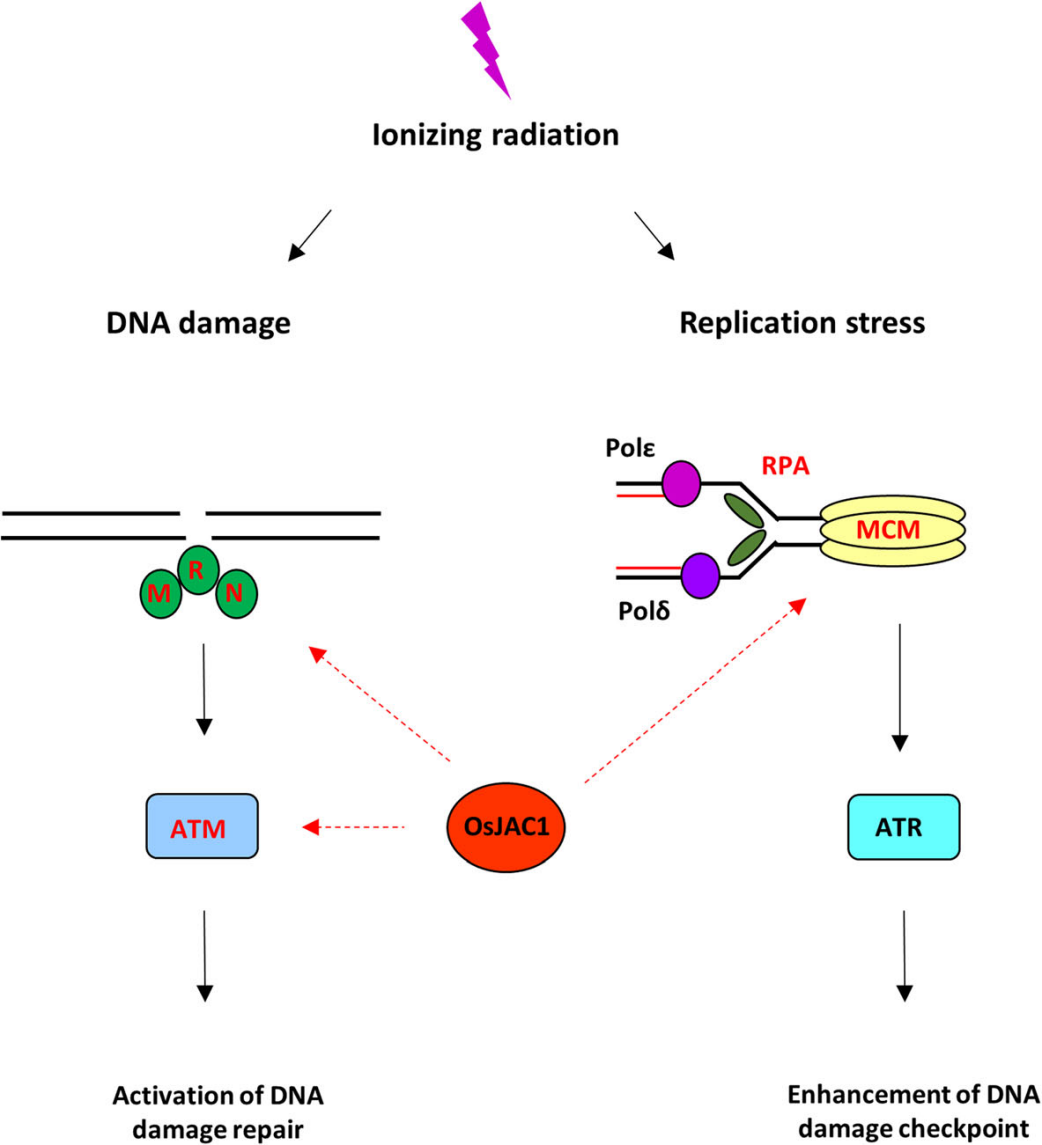
Scheme of the involvement of OsJAC1 in the DDR pathway. Dotted lines indicate possible regulation or transcriptional coordination of the pathway by OsJAC1

Figure 9 displays a scheme illustrating the hyper-resistance to ionizing radiation conferred by OsJAC1 overexpression. In summary, we suggest that the observed upregulation of *ATM* and *MRE11* by OsJAC1 overexpression provides evidence of enhanced DNA damage perception. We interpret the observed transcriptional changes of genes encoding DNA polymerases, RPAs, and MCMs as evidence for the activation of DNA damage checkpoints in response to replication stress in OsJAC1-overexpressing lines. Thus, activation of both DNA damage perception and DNA damage checkpoints by OsJAC1 overexpression may confer hyper-resistance to gamma radiation in *Arabidopsis*.

## Methods

### Plant growth conditions

*Oryza sativa spp. japonica* cv. Ilpoom was obtained from the Rural Development Administration of Korea. *Arabidopsis thaliana* ecotype *Landsberg erecta*, originated from the Arabidopsis Biological Resource Center, was acquired from Kumho Life Science Laboratory of Chonnam National University in Korea. Rice plants were grown at 30 °C with a cycle of 16 h light followed by 8 h dark. *Arabidopsis* plants were cultured at 23 °C under the light and dark cycle as described above.

### Generation of OsJAC1-overexpressing *Arabidopsis* lines

*OsJAC1* (XM_015763269) cDNA was amplified with gene-specific primers using the polymerase chain reaction (PCR). The PCR conditions were as follows: one cycle at 94 °C for 5 min; 35 cycles at 92 °C for 1 min, 57 °C for 1 min, and 72 °C for 1 min; and one cycle at 72 °C for 5 min. Primer sequences for *OsJAC1* were 5′-ATG GCT GAT CCC AGC AAG CTG CA-3′ and 5′-TTA GAT CGG CTG CAC GTA GAC ACC AAC-3′. The amplified *OsJAC1* cDNA was sub-cloned into the pCR™8/GW/TOPO® vector and then transferred into the pMDC83 vector using the Gateway cloning system according to the manufacturer’s instructions. The OsJAC1-overexpressing construct was introduced into *Agrobacterium tumefaciens* LBA4404 using electroporation. *Arabidopsis* plants were transformed using the floral dip method [64]. Seeds were harvested from the dipped *Arabidopsis* plants. To identify insertion of the OsJAC1-overexpressing construct, selection was performed using MS media containing 50 μg/ml kanamycin. To obtain homozygous OsJAC1-overexpressing lines, segregation analyses of seeds from the selected progenies were carried out. Six homozygous lines with OsJAC1 overexpression were identified.

### Conditions of gamma irradiation

Rice seeds were germinated on Murashige and Skoog (MS; Duechefa, Haarlem, Netherlands) solid media containing 0.8% agar and 1% sucrose. Two-week-old seedlings were irradiated with gamma radiation using a gamma irradiator (^60^Co, approximately 150 TBq; Atomic Energy of Canada, Ltd., Ottawa, Ontario) for 12 h at the Korea Atomic Energy Research Institute. To identify dose-dependent effects, various doses (100, 200, 300, and 400 Gy) of gamma radiation were used for each sample. Seedling samples were obtained at different times (0–168 h) after gamma irradiation for analysis. For confirmation of time-dependent expression of *OsJAC1* in response to ionizing radiation, dry rice seeds were exposed to gamma radiation at different doses (100, 200, 300, and 400 Gy), and then seeds were germinated on MS media. Two-week-old rice seedlings were harvested.

### Imposition of salinity stress and treatment with plant hormones

For plant hormone treatment, rice seeds were germinated in MS solid media containing 0.8% agar and 1% sucrose. Two-week-old rice seedlings were treated with 1 mM SA (Sigma, St. Louis, MO, USA) and 0.1 mM JA (Sigma). Samples were collected at 6, 12, and 24 h after each treatment. For imposition of heat stress, 2-week-old rice seedlings were incubated at 45 °C for 2 h. Samples were obtained 0, 3, 6, and 12 h after heat treatment.

### RNA isolation and quantitative reverse transcription (RT)-PCR

Total RNA was isolated using RNeasy plant mini kit (Qiagen, Hilden, Germany) according to the manufacturer’s instructions, and then DNA contamination was removed using RNase-free DNase (Takara, Kyoto, Japan). The cDNA synthesis was performed using the Superscript®III reverse transcriptase (Invitrogen, Carlsbad, CA, USA). For quantitative RT-PCR, cDNA amplification was performed using Power SYBR Green PCR master mix (Thermo Fisher Scientific, Rockford, IL, USA) with the CFX™ Real-Time System (Bio-Rad, Hercules, CA, USA). Conditions for the PCR reactions were as follows: one cycle at 94 °C for 5 min; 40 cycles at 92 °C for 30 s, 60 °C for 30 s, and 72 °C for 30 s; one cycle at 72 °C for 5 min. Primer sequences for *OsJAC1* were 5′-CGT CTC GAA AGC ATC ACA TT-3′ and 5′-CGG CAT GGT CAA GGT AAG TA-3′ and for *Actin* were 5′-TGA AGT GCG ACG TGG ATA TTA G-3′ and 5′-CAG TGA TCT CCT TGC TCA-3′.

### Western blot analysis

For total protein extraction, whole plant tissues were homogenized in extraction buffer (100 mM Tris-Cl, pH 7.5; 1 mM ethylenediaminstetraacetic acid; 0.5 NP-40; 150 mM NaCl; 3 mM dithiothreitol) and protease inhibitor (Sigma). Total proteins were separated on a sodium dodecyl sulfate-polyacrylamide gel (Sigma) by electrophoresis and then transferred onto Immobilon-P membranes (Millipore, Burlington, MA, USA). Immunodetection was performed with a rat anti-GFP antibody (Abcam, Cambridge, MA, USA) and visualized using a chemiluminescence ECL kit (Thermo Fisher Science, Waltham, MA, USA) according to the manufacturer’s instruction.

### Comparative transcriptome analysis

Two biological plant sample replicates were prepared for transcriptome analysis. RNA isolation was performed as described above. Transcriptome analysis was conducted as described by Koo et al. [65]. Briefly, mRNA-Seq paired-end libraries were constructed using the Illumina TruSeq RNA Sample Preparation Kit v2 (Illumina, San Diego, CA, USA), and the KAPA library quantification kit (Kapa Biosystems, Wilmington, MA, USA) was utilized for quantification of the library according to the manufacturer’s instruction. The cDNA libraries were sequenced using an Illumina HiSeq2000 (Illumina). For short-read mapping, reads were mapped to reference transcripts using the bowtie software (Langmead et al., 2009). DEGs (*p* ≤ 0.01 and fold-change ≥2) commonly expressed between the transgenic lines in comparison with the control were selected from the mapped reads.

### Statistical analyses

One-way analyses (ANOVA) were performed for statistical analyses of quantitative RT-PCR and plant growth measurement using R program (version 3.6.1).

## Supplementary information

**Additional file 1: Figure S1.** Root growth of OsJAC1-overexpressing plants in response to salt stress.

**Additional file 2: Table S1.** Expression levels of anotated transciprts in OsJAC1-overexpressing *Arabidopsis* lines.

## Data Availability

All materials in the current article are available from the corresponding author.

## Abbreviations

ATM: Ataxia Telangiectasia Mutated protein
DDR: DNA damage response
DEGs: Differentially expressed genes
DSB: Double-strand breaks
OsJAC1: Rice mannose-binding jacalin-related lectin
