# Supplementary material for: Overexpression of rice jacalin-related mannose-binding lectin (OsJAC1) enhances resistance to ionizing radiation in Arabidopsis
Source: BMC Plant Biol. 2019 Dec 18;19:561. doi: 10.1186/s12870-019-2056-8 (PMC6921557; doi:10.1186/s12870-019-2056-8)
Supplement: Supplementary file 1 — Additional file 1: Figure S1. Root growth of OsJAC1-overexpressing plants in response to salt stress. [file 12870_2019_2056_MOESM1_ESM.pptx]

## Slide 1
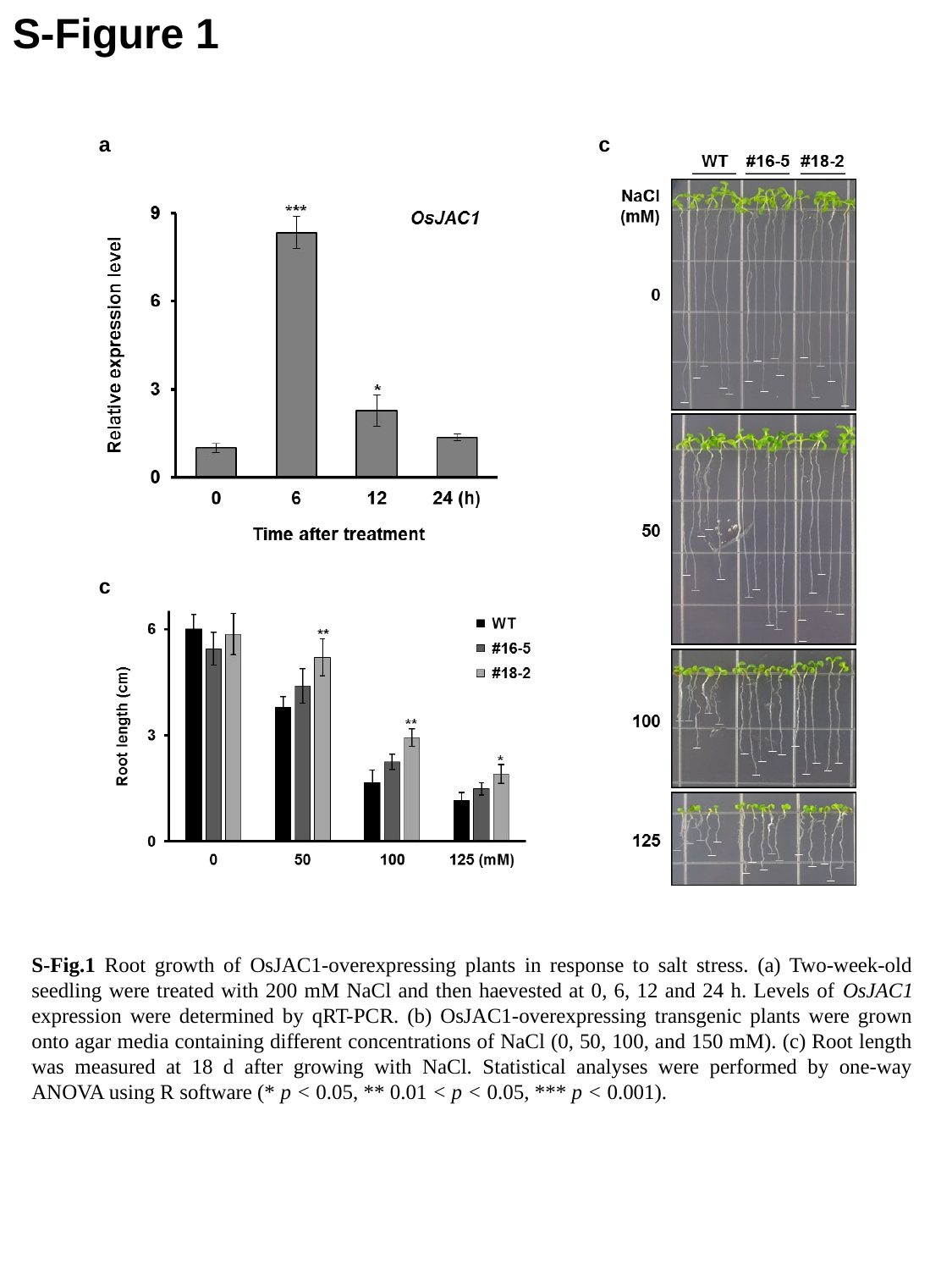

S-Figure 1
a
c
c
S-Fig.1 Root growth of OsJAC1-overexpressing plants in response to salt stress. (a) Two-week-old seedling were treated with 200 mM NaCl and then haevested at 0, 6, 12 and 24 h. Levels of OsJAC1 expression were determined by qRT-PCR. (b) OsJAC1-overexpressing transgenic plants were grown onto agar media containing different concentrations of NaCl (0, 50, 100, and 150 mM). (c) Root length was measured at 18 d after growing with NaCl. Statistical analyses were performed by one-way ANOVA using R software (* p < 0.05, ** 0.01 < p < 0.05, *** p < 0.001).
